# Therapeutic Effects in a Transient Middle Cerebral Artery Occlusion Rat Model by Nose-To-Brain Delivery of Anti-TNF-Alpha siRNA with Cell-Penetrating Peptide-Modified Polymer Micelles

**DOI:** 10.3390/pharmaceutics11090478

**Published:** 2019-09-15

**Authors:** Takanori Kanazawa, Takumi Kurano, Hisako Ibaraki, Yuuki Takashima, Toyofumi Suzuki, Yasuo Seta

**Affiliations:** 1 School of Pharmacy, Tokyo University of Pharmacy and Life Sciences, 1432-1 Horinouchi, Hachioji, Tokyo 192-0392, Japan; phta19001@g.nihon-u.ac.jp (T.K.); ibaraki@toyaku.ac.jp (H.I.); takasima@toyaku.ac.jp (Y.T.); setayas@toyaku.ac.jp (Y.S.); 2 School of Pharmacy, Nihon University, 7-7-1 Narashinodai, Funabashi, Chiba 274-8555, Japan; suzuki.toyofumi@nihon-u.ac.jp

**Keywords:** nose-to-brain delivery, siRNA, cell-penetrating peptide, polymer micelle, transient middle cerebral artery occlusion, cerebral ischemia-reperfusion injury

## Abstract

We previously reported that siRNA delivery to the brain is improved by the nose-to-brain delivery route and by conjugation with polyethylene glycol-polycaprolactone (PEG-PCL) polymer micelles and the cell-penetrating peptide, Tat (PEG-PCL-Tat). In this study, we evaluated the nose-to-brain delivery of siRNA targeting TNF-α (siTNF-α) conjugated with PEG-PCL-Tat to investigate its therapeutic effects on a transient middle cerebral artery occlusion (t-MCAO) rat model of cerebral ischemia-reperfusion injury. Intranasal treatment was provided 30 min after infarction induced via suturing. Two hours after infarction induction, the suture was removed, and blood flow was released. At 22 h post-reperfusion, we assessed the infarcted area, TNF-α production, and neurological score to determine the therapeutic effects. The infarcted area was observed over a wide range in the untreated group, whereas shrinkage of the infarcted area was observed in rats subjected to intranasal administration of siTNF-α with PEG-PCL-Tat micelles. Moreover, TNF-α production and neurological score in rats treated by intranasal administration of siTNF-α with PEG-PCL-Tat micelles were significantly lower than those in untreated and naked siTNF-α-treated rats. These results indicate that nose-to-brain delivery of siTNF-α conjugated with PEG-PCL-Tat micelles alleviated the symptoms of cerebral ischemia-reperfusion injury.

## 1. Introduction

Cerebral strokes are broadly classified either as cerebral infarctions, where the cerebral artery is constricted or obstructed causing necrosis in surrounding tissues, or as cerebral hemorrhages such as intracranial hemorrhages and subarachnoid hemorrhages [1,2]. Conventional pharmacotherapies for ischemic brain injuries are broadly classified into four types: thrombolytic therapy, brain protection therapy, antiplatelet therapy, and anticoagulant therapy. In the treatment guidelines for cerebral stroke, particularly for the acute phase, thrombolytic therapy—intravenous administration of tissue plasminogen activator (t-PA) within 4.5 h of the blockage—is strongly recommended [3]. However, there are still a variety of other issues remaining to be solved, such as the fact that this type of treatment is only used in approximately 3–8% of all cases [4,5].

In recent years, enlargement of the infarcted area and worsening of symptoms due to the post-ischemic inflammatory response have been focused upon [2,6]. Development of treatments that suppress post-ischemic inflammation and protect cranial nerves holds a lot of promise. Various inflammatory cytokines have been suggested as potential targets for such treatments [7,8]. The disruption of the blood–brain barrier (BBB) after stroke is associated with an inflammatory response and impaired cerebral autoregulation. First, HMGB1 is released from brain neurons that have entered ischemia, and BBB breaks down by acting on cerebral vascular endothelial cells. In addition, nerve cell also releases peroxiredoxins, and macrophages is infiltrated. As a result, inflammation during ischemia occurs due to the production of inflammatory cytokines such as TNFα, IL-1β, and NF-κB from macrophages [9,10,11].

Nucleic acid medicines hold great promise as a next-generation modality that can target new molecules such as intracellular mRNA. For central nervous system diseases, Spinraza^®^ was approved in the US in 2016 and was subsequently approved in Europe/Japan to treat myelopathic muscular atrophy [12,13]. Nucleic acid medicines show increasing promise as candidate drugs against central nervous system diseases. However, these biopharmaceuticals are significantly limited to transport to the central nervous system. This is because the blood-brain barrier (BBB), which is made up of tight junctions between vascular endothelial cells and the astrocytic foot processes surrounding them, prevents entering to the brain of pharmaceuticals. Due to this, the development of drug delivery systems to bypass the BBB is key to the development of therapeutics for central nervous system diseases. Further, on the heels of antisense nucleic acids, small interfering RNA (siRNA) holds promise as a nucleic acid medicine for clinical applications as it has a larger molecular weight than antisense nucleic acids and a double-stranded linear structure. Therefore, it is necessary to develop nanocarriers such as lipid nanoparticles and polymer micelles, in addition to chemical modification technologies.

In recent years, nasal administration focusing on the connection between the nose and brain has attracted focus in research on drug delivery to the brain because it is possible to use nasal administration to deliver drugs to the brain quickly via a unique route [14,15,16,17,18,19]. This route holds promise in avoiding drug elimination mechanisms in the blood circulating throughout the body and in alleviating side effects. Moreover, self-administration is possible, and it may be highly useful as a minimally invasive treatment in human. Many studies have investigated the nasal administration of low-molecular-weight drugs as well as medium and high-molecular-weight drugs such as peptides, proteins, and nucleic acid therapeutics. However, due to low penetration of medium and high-molecular-weight drugs across the nasal epithelial layer into lamina propria which connect to nose-to-brain delivery pathways after nasal administration, controlling the amount of them that can be transported and its concentration in the brain is difficult; therefore, development of a nose-to-brain drug delivery system that penetrates this barrier is required [20,21,22].

We have previously designed a nose-to-brain drug delivery system consisting of membrane-penetrating polymer micelles composed of PEG-PCL-Tat, which contains PEG-PCL (that is self-assembling in water) conjugated with a cell membrane-penetrating peptide [23,24,25,26,27,28,29]. We attempted the delivery of siRNA, a double-stranded RNA that exerts a gene-silencing effect by cutting the sequence-specific target mRNA through RNA interference, by loading it onto these PEG-PCL-Tat micelles for intranasal administration for delivery to the brain [25,26]. We have reported that it is possible to use PEG-PCL-Tat micelles for efficient penetration of the nasal mucosa and for transporting siRNA to the brain [25]. Furthermore, applying this nose-to-brain delivery system using PEG-PCL-Tat micelles to brain tumor model rats, we also demonstrated excellent inhibition of brain tumor growth and a life-prolonging effect [24,26].

In the present study, we aimed to apply membrane-penetrating peptide-modified polymer nanomicelles for the treatment of ischemic brain injury via nasal administration. We chose siRNA targeted for TNF-α for reduction of TNF-α expression expecting neuroprotective effect and complexed with PEG-PCL-Tat. Then, we investigated its therapeutic effect in transient ischemic reperfusion (t-MCAO) model rats.

## 2. Materials and Methods

### 2.1. Materials, Cells, and Rats

The anti-TNF-α siRNA (siTNF-α; sense, 5′-AAGAGAACCUGGGAGUAGAUAAGGUdTdT -3′) and Fluorescence 6-carboxyfluorescein-aminohexylphosphoramidite (FAM)-labeled siRNA (FAM-siRNA), were obtained from Cosmo Bio Co., Ltd., (Tokyo, Japan). Methoxy polyethylene glycol (Mn: 2000)-polycaprolactone (Mn: 2000) (PEG-PCL) was purchased from Sigma-Aldrich Co. (Milwaukee, WI, USA). Tat-G (GlyArgLysLysArgArgGlnArgArgArgGly) was obtained from BEX Co., Ltd. (Tokyo, Japan). Rat TNF-α ELISA kit was purchased from R&D Systems, Inc. (Minneapolis, MN, USA)

The rat neuronal RN33B cell lines were purchased from American Type Culture Collection (Manassas, VA, USA). Dulbecco’s modified Eagle medium/F12 (1:1) (DMEM/F12, Life Technologies Japan Ltd., Tokyo, Japan), certified fetal bovine serum (FBS, Life Technologies Japan Ltd., Tokyo, Japan), penicillin/streptomycin stock solutions (Thermo Fisher Scientific K.K., Tokyo, Japan), N2 supplement (Thermo Fisher Scientific K.K., Tokyo, Japan), 0.25% Trypsin-EDTA (Life Technologies Japan Ltd., Tokyo, Japan), Lipotrust™ (Hokkaido System Science, Sapporo, Japan), and cell counting kit-8 (CCK-8, Dojindo Laboratories, Kumamoto, Japan) solution were used for the cellular uptake and cytotoxicity assays in RN33B cells.

Ten-week-old Sprague–Dawley (SD) male rats were purchased from Japan SLC Inc. (Shizuoka, Japan). The rats were housed under standard conditions of temperature (22–24 °C) and humidity (40–60%) under a 12-h light/12-h dark cycle with the light period starting at 08:00. Food and water were supplied ad libitum. All the animal experiments were performed in accordance with a protocol of P17-80 (April, 2017) and P18-07 (April, 2018) approved by the Animal Care and Ethics Committee of Tokyo University of Pharmacy and Life Sciences.

### 2.2. Synthesis of a Tat Analog Conjugated with PEG-PCL through a Disulfide Linkage

The PEG-PCL-Tat was synthesized according to our previous study [23,24,25,26,30]. In general, Tat-G and PEG-PCL were dissolved in DMF. Water soluble carbodiimide hydrochloride (WSCI) were added to this mixture and allowed to react at room temperature for 24 h, causing the Gly-COOH on the C terminus of Tat-G and the terminal –OH group on PEG-PCL to form an ester bond. The reaction solution was transferred to a dialysis membrane suitable for use with organic solvents (Spectra/Por^®^ Dialysis Membranes, MWCO: 3500) and dialyzed against distilled water for 3 days. Afterwards, the product was lyophilized to yield PEG-PCL-Tat.

### 2.3. Preparation of the siRNA/PEG-PCL-Tat Complex

Solutions of siTNF-α or FAM-siRNA were mixed by steadily dripping them into PEG-PCL-Tat solution so that the N/P ratio was between 5 and 30, then left to stand for 30 min, resulting in the formation of siRNA/PEG-PCL-Tat complexes.

### 2.4. Physicochemical Characterization of siRNA/PEG-PCL-Tat Complexes

The mean particle size and zeta potential of siTNF-α/PEG-PCL-Tat complexes with an N/P ratio of 5–30 were measured using a dynamic light scattering photometer DLS-700 unit (Otsuka electronics Co., Ltd., Osaka, Japan) and NICOMP 380ZLS unit (Particle Sizing Systems, Shanghai, China), respectively.

SYBR Green solution was added to the siTNF-α solution. After 30 min of incubation, the PEG-PCL-Tat solution with an N/P ratio from 5 to 30 was added to the siTNF-α solution. The fluorescence of each sample was then measured after 30 min using a microplate reader (Safire Microplate Reader, TECAN, Japan) at Ex: 485 nm, Em: 538 nm. The fluorescence of naked siTNF-α was 100%.

### 2.5. Evaluation of siRNA Cellular Uptake Ability in RN33B Neuronal Cells

RN33B cells (2 × 10^5^) in 12-well culture plates were differentiated to neuron cells in DMEM/F12 with 10% FBS and 1% N2 supplements at 33 °C in a humidified 5% CO_2_ atmosphere as preculture. The passage number range was from 5 to 7. After 48-h incubation, the cells were washed with PBS and transfected with naked FAM-siRNA, FAM-siRNA/Lipotrust complex as a positive control or FAM-siRNA/MEG-PCL-Tat. The FAM-siRNA concentration in all samples was 1 µg/well. Four hours after transfection, the RN33B cells were washed with PBS and their uptake capacity was measured with a flow cytometer (BD FACS Canto; BD Biosciences, Franklin Lakes, NJ, USA). The cell population is gated on the plot of the side scattered light with respect to the forward scattered light. In the plot of the cell number relative to the fluorescence intensity in the control of the target cell group, the range including 95% of the cells is defined as the P1 region (10,000 cells) and the range in which the fluorescence intensity is stronger is defined as the P2 region. Thus, the percentage of cells in the P2 region was used as an indicator of siRNA uptake into cells. Additionally, the fluorescence intensity per cell was calculated by the average value of fluorescence intensity in the P2 region as the cellular uptake efficiency.

### 2.6. In Vitro Cytotoxicity of PEG-PCL-Tat in RN33B Neuronal Cells

RN33B cells (50,000 per well) in 200 µL DMEM/F12 with 10% FBS were seeded onto 96-well culture plates and differentiated for 48 h-incubation at 33 °C in a humidified 5% CO2 atmosphere. After incubation, the cells were washed with PBS and transfected with PEG-PCL-Tat of equivalent concentration to various N/P ratio (10, 30, 50) for siRNA concentration of 0.2 µg/200 µL. After 4 h, the cells were washed with PBS, and CCK-8 solution was added to each well followed by incubation for 3 h. The absorbance of each well was measured with a microplate reader (Safire Microplate Reader, Tecan) at 450 nm. The absorbance of the control cells was set as 100% viability. The viability of all cells was expressed as a percentage relative to the absorbance of the control cells.

### 2.7. Creating Transient Middle Cerebral Artery Occlusion (t-MCAO) Model Rats by Filamentous Occlusion

For filamentous occlusion, 4.0 monofilament nylon thread (Ethicon, Johnson and Johnson, Somerville, NJ, USA) was used. The 4.0 monofilament nylon thread was cut to a length of 3.5 cm, roughly 2–3 mm sections at the ends were coated with a mixture of Provil^®^ novo Base and Provil^®^ novo Catalyst and were placed in an incubator for 24 h. Once the monofilament was completely dried, the ends were further coated with JM Silicone adhesive. The diameter of the portion forming the occlusion was adjusted to 0.39 mm by comparison with a 27G needle under a microscope. The monofilament was again placed in an incubator for 24 h to dry it completely.

Next, 300 g male SD rats were anesthetized with pentobarbital (50 mg/kg) via intraperitoneal injection. The rats were fixed on a cork board with the animal’s body in a face up position. The neck of the animal was wiped with ethanol for disinfection, and the hair was shaved with hair clippers. Next, 0.2 mL of 0.5% bupivacaine was administered to the neck by subcutaneous injection. An incision was made in the neck and the common carotid artery (CCA) was located and isolated with tweezers. Following this, the branching into the external carotid artery (ECA) and internal carotid artery (ICA) was located and isolated. Then, the superior thyroid artery (STA) and occipital artery (OA), which are found to branch off near the ECA, were cauterized via bipolar electrocautery (SX-2001, Tagawa Electricity Research Laboratories, Ltd., Chiba, Japan). Furthermore, after locating the pterygopalatine artery (PPA) branch point on the ICA, the PPA was cauterized. Then, the ECA was sutured, and artery clamps were placed on the CCA followed by the ICA. A half incision was made in the sutured ECA, and the prepared nylon suture was inserted. The artery clamp fixed on the ICA was removed, and the middle cerebral artery of the rat was occluded by further inserting the nylon suture up to the 20–23 mm mark from its end. The artery clamp fixed on the CCA was removed, and after confirming the absence of hemorrhaging, the cut-open neck was stitched back together.

After the middle cerebral artery occlusion caused ischemia for 2 h, the nylon suture was removed, reopening the blood vessel and creating a transient ischemia-reperfusion injury model rat. After again cutting open the neck where it was sewed shut, the occluded portion up to the incised part of the ECA where the nylon suture was inserted was extracted, and artery clamps were fixed onto the ICA and CCA in that order. After the nylon suture was completely extracted and the ECA was completely sutured, the artery clamps were removed, thus reopening blood flow in the rat’s middle cerebral artery. The surgical incision was again sewn shut and sterilized, and reperfusion was allowed to occur for 22 h.

### 2.8. Intranasal Administration to t-MCAO Model Rats

The nasal administration was performed according to our previous study [19,23,24,25,26,27,28]. At 30 min after middle cerebral artery occlusion in t-MCAO model rats, the rats were immobilized under pentobarbital anesthesia in a face up position so that their nose and neck were horizontally oriented. The nasal administration used for 1–10 μL micropipette attached with the disposable polypropylene tips. The prepared sample solution was transferred to each nostril using micropipettes, 2 μL at a time for a total of 80 μL containing 30 μg of siTNF-α, administering it via each nostril alternately at every 30 s.

### 2.9. Evaluation of Neurology Score

The neurology score was evaluated as an indicator of treatment efficacy according to previous paper [31]. The neurology score of rats at 22 h after reperfusion was given based on spontaneously activity, drifting during displacement, parachute reflex, and resistance to right forepaw stretching, each of which has fixed standards.

### 2.10. Brain Extraction

Under anesthesia via intraperitoneal injection of pentobarbital (50 mg/kg), a median incision was made in the scalp down to the occipital region, exposing the posterior surface of the occipital region, first cervical vertebra, and second cervical vertebra. Next, an incision was made between the first and second cervical vertebrae using scissors to euthanize the rat. In the cut head, the skull was cut down the median with scissors from the occipital bone to the frontal bone. The skull up to the temporal bone was removed to the greatest extent using tweezers, to expose the brain. The dura mater was removed; the optical nerve, trigeminal nerve, and so on were cut, and the brain was extracted. The extracted brains were used to measure the occlusion rate or the concentration of TNF-α.

### 2.11. Observation of the Infracted Area via TTC Staining Continuous Coronal Brain Slice and Calculation of Infracted Area (%)

The isolated brain was sliced in the coronal direction at 2-mm intervals starting from the tip of the brain using a tissue-slicer. The posterior side of the continuous coronal brain slice sections was placed down in a Petri dish filled with a 2% 2,3,5-triphenyltetrazolium chloride (TTC) solution, allowed to sit in the dark in an incubator for 15 min, and thus stained. The stained continuous coronal brain slices were imaged, and the ratio of the total area of 6 continuous coronal brain slices from the tip of the brain, which had the olfactory bulb removed, to the proportion of the area in the sections that were infarcted was used to calculate the infracted area (%) using the Image J image analysis program.

### 2.12. Measurement of TNF-α Concentration in the Brain

After the olfactory bulb and cerebellum were cut from the extracted brain, the brain was separated into the left brain and the right brain. Next, 5 mL/g tissue of lysis buffer was added to the left brain. Lysis buffer was composed by Ethylenediamine-*N*,*N*,*N*’,*N*’-tetra acetic acid (EDTA), disodium salt di-hydrated (DOJINDO laboratories, Kumamoto, Japan), Triton×100 (Sigma-Aldrich Co., Milwaukee, WI, USA), Tween 80 (FUJIFILM Wako Chemicals, Osaka, Japan), NaCl (FUJIFILM Wako Chemicals, Osaka, Japan), and Tris-HCl (pH 7.5) (Nippon gene, Tokyo, Japan) in distilled water. The left brain was cut into fine pieces with scissors and homogenized at 10,000 rpm for 3 min in a homogenizer (polytron^®^ PT3100, Kinematica, A.A.; Luzern, Switzerland). The homogenized liquid was fractioned into a 1-mL tube, which was then centrifuged for 30 min at 12,000 rpm. Then, 400 μL of the supernatant was used as a sample solution to determine the concentration of TNF-α. The sample was stored at −20 °C until measurement. TNF-α concentration was measured in accordance with the protocol of the ELISA kit. The limit of detection range for rat TNF-α ELISA kit was 12.5–800 pg/mL.

### 2.13. Statistical Analysis

The statistical analysis was performed byBellCurve for Excel (Social Survey Research Information Co., Ltd., Tokyo, Japan). The results of the experiments are represented as the mean ± S.E. Comparisons between multiple treatments were made using analysis of variance (ANOVA), followed by Dunnett’s test. Group differences were considered statistically significant when *P* < 0.05. Statistical significance was defined as * *p* < 0.05 and ** *p* < 0.01.

## 3. Results

### 3.1. Evaluation of Physical Properties of the siTNF-α/PEG-PCL-TAT Complex

**Figure 1:**
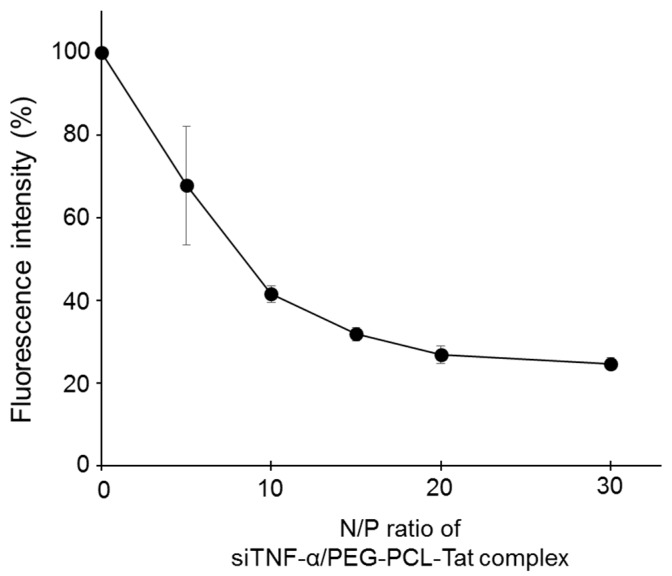
Complex formation ability with siTNF-α by PEG-PCL-Tat micelles. Complex formation ability with siTNF-α were determined using SYBR Green Exclusion Assay. The fluorescence of siTNF-α and PEG-PCL-Tat complexes at several N/P ratios from 0 to 30 were measured using a microplate reader. Each point represents the mean ±S.D. (*n* = 3).

Figure 1 shows a SYBR^®^Green exclusion assay to evaluate the ability of siTNF-α/PEG-PCL-Tat complex formation. Considering the fluorescence of naked siTNF-α as 100%, the relative fluorescence decreased with increasing N/P ratio. This is thought to be because there is a reduction in the degree of SYBR^®^Green intercalation in the siRNA due to a complex forming between siTNF-α and PEG-PCL-Tat. The more the N/P ratio increases, the more rigid is the complex formed. From the Figure 1, we observed that fluorescence intensity decreases starting from an N/P ratio of 15. This indicates that PEG-PCL-Tat with an N/P ratio of 15 or more has a high siRNA condensation ability.

Then, Table 1 shows that the particle size and zeta potential of siTNF-α/MPEG-PCL-Tat complexes. The particle size of these MPEG-PCL-Tat/siTNF-α complexes ranged from 50 to 100 nm, and the particle size decreased with an increase in the N/P ratio. The siTNF-α/PEG-PCL-Tat complex carries a positive charge for N/P ratios of 5 and more; the electrical potential increased as the N/P ratio increased. This is thought to be due to the Tat peptide being exposed on the surface of the complex because of an increase in the amount of PEG-PCL-Tat.

### 3.2. siRNA Cellular Uptake and Cytotoxicity by PEG-PCL-Tat in RN33B Neuronal Cells

**Figure 2:**
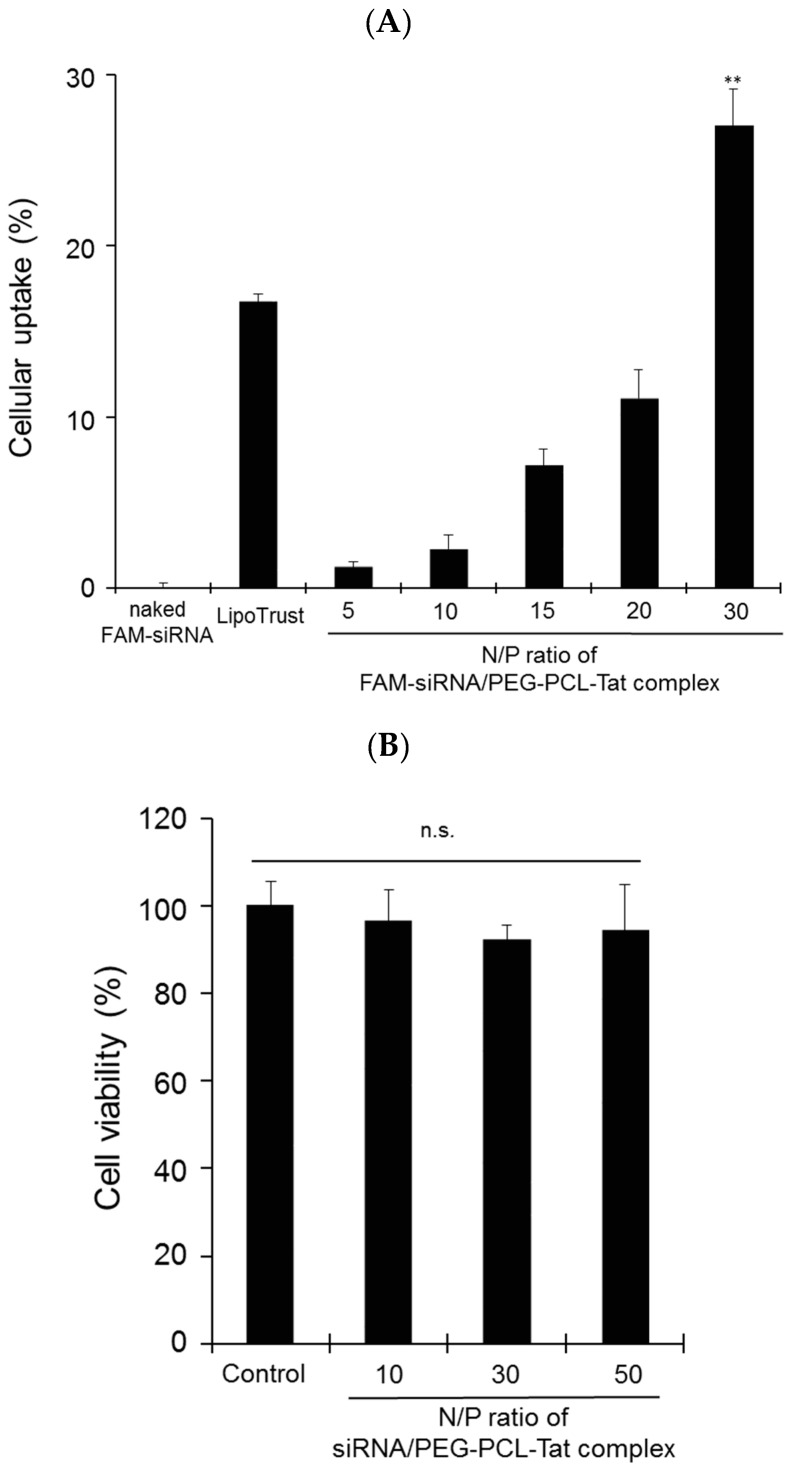
Cellular uptake of siRNA and cytotoxicity by PEG-PCL-Tat micelles in rat RN33B neuronal cells. RN33B cells were transfected with naked FAM-siRNA (1 μg), FAM-siRNA (1 μg) complexed with PEG-PCL-Tat (N/P ratio: 5–30), or Lipotrust as positive control. (**A**) After incubation for 4 h, the cellular uptake (%) of FAM-siRNA into RN33B cells was determined by flow cytometry. (**B**) After incubation for 3 h, in vitro cytotoxicity by PEG-PCL-Tat was determined by WST-8 assay. Each bar represents the mean ± S.D. (*n* = 4). ** *p* < 0.01 vs. other groups, ^n.s.^ *p* > 0.05.

Figure 2 shows the intracellular uptake (Figure 2A) and cytotoxicity (Figure 2B) in RN33B neuronal cells that were made to differentiate into nerve cells when transfected with PEG-PCL-Tat corresponding to each N/P ratio.

Based on the Figure 2, it is clear that PEG-PCL-Tat has low cytotoxicity and a good ability to form complexes with siRNA and that 100 nm or smaller stable particles that carry a positive charge accompanying an increase in the N/P ratio are formed. We used an N/P ratio of 30 for the subsequent experiment as it had the highest complex-forming ability.

### 3.3. Treatment Effects for Transient Ischemia-Reperfusion Injury Treatment Efficacy Using t-MCAO Model Rats

**Figure 3:**
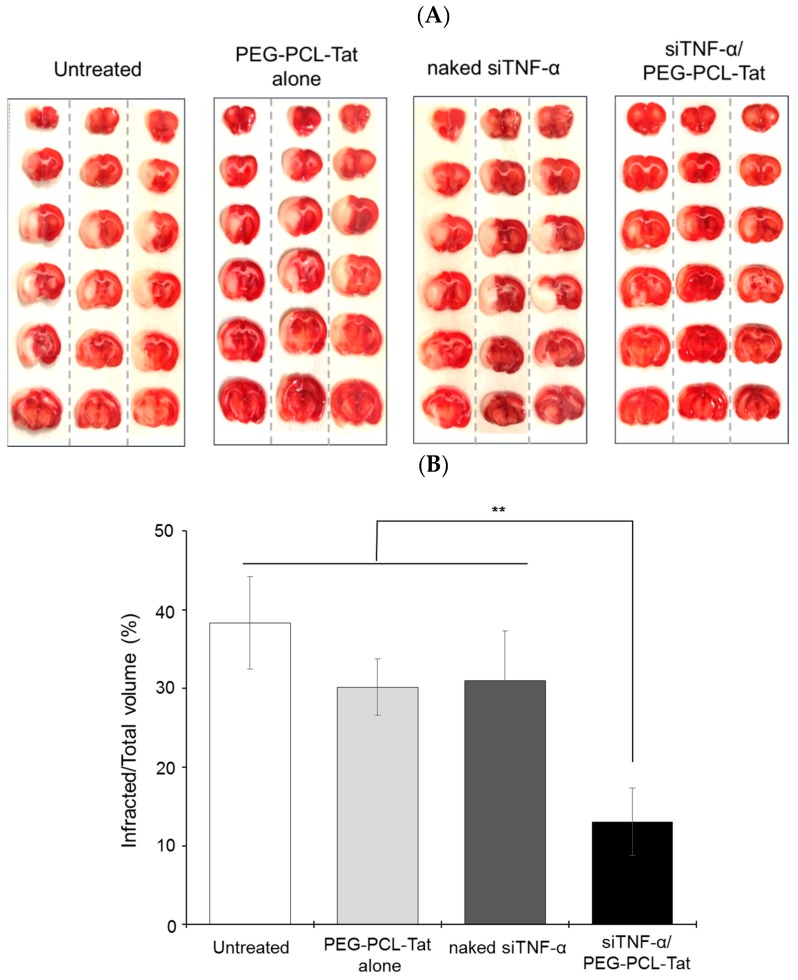
The representative Images of the TTC staining of continuous coronal brain slice and infracted area (%) in MCAO rats treated with intranasal administration of siTNF-α/PEG-PCL-Tat complex. (**A**) Each brain from MCAO rats in each treating group was isolated, and each isolated brain was sliced in the coronal direction at 2-mm intervals. The continuous coronal brain slice sections were stained with a 2% TTC solution. (**B**) The infracted area (%) for a total area of 6 continuous coronal brain slices were calculated using the Image J image analysis program. Each bar represents the mean ± S.E. (*n* = 5). ** *p* < 0.01.

Figure 3 shows images of continuous coronal brain slice sections after TTC staining (Figure 3A), and the results of the calculations for the infarct area proportion using the Image J image analysis program (Figure 3B).

In the untreated group, the left brain in which the infarct was caused had a widespread white infarcted area in all continuous coronal brain slice sections. Moreover, the PEG-PCL-Tat-only treatment group and the siRNA-only treatment group both showed virtually the same results as the untreated group. In contrast, the infarcted area was significantly reduced in the siTNF-α/PEG-PCL-Tat complex administration group. Moreover, for the siTNF-α/PEG-PCL-Tat administration group, the increase in the infarcted proportion was significantly suppressed based on the area of the infarcted location calculated by Image J compared to the untreated group, the PEG-PCL-Tat-only group, and the siRNA-only group. Since the suppression effect on the expansion of the infarcted area was not observed in the PEG-PCL-Tat-only group, it is clear that PEG-PCL-Tat itself does not have a therapeutic effect. Moreover, the siTNF-α single administration group did not show any treatment efficacy possibly because the naked siRNA was degraded due to low stability in vivo, such as in the nasal mucosa. Moreover, the siRNA may not be able to well permeate the nasal mucosa and is not transported to the brain.

**Figure 4:**
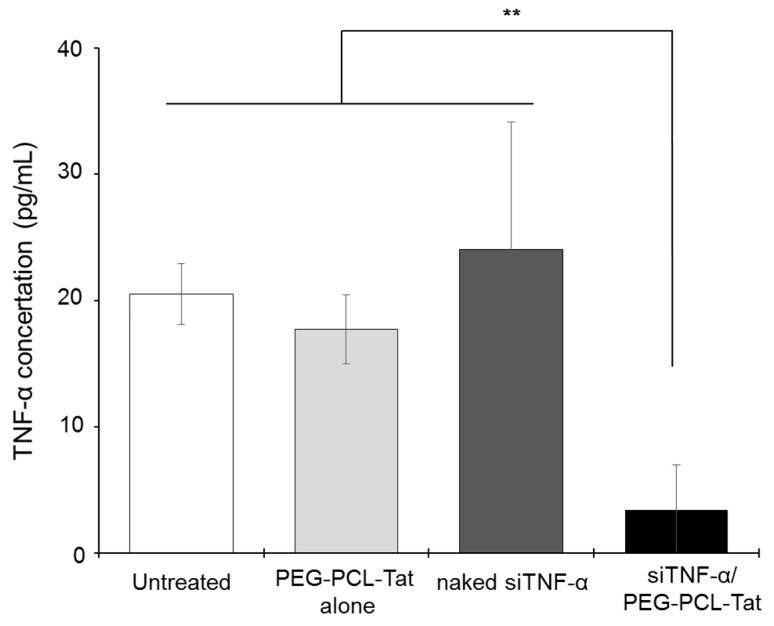
The TNF-α concentration in the brain of MCAO rats treated with intranasal administration of siTNF-α/PEG-PCL-Tat complex. Each left brain from MCAO rats in each treating group was isolated and homogenized. The TNF-α concentration in the supernatant of centrifuged homogenate was determined was measured by ELISA kit. Each bar represents the mean ± S.E. (*n* = 5). ** *p* < 0.01.

Figure 4 shows the results of TNF-α production in the infarcted tissue using ELISA. The graph indicates that the PEG-PCL-Tat-only treatment and siRNA-only treatment groups showed no suppression of TNF-α production, similar to that in the untreated group. In contrast, the siTNF-α/PEG-PCL-Tat complex group showed drastically and significantly suppressed TNF-α production compared to the other groups.

The above results suggest that nasal administration of the siTNF-α/PEG-PCL-Tat complex increases the nose-to-brain delivery of siTNF-α via CPP, and increases the nerve cell uptake of siRNA, thus drastically suppressing the expression of TNF-α in the brain. Moreover, as the nasal administration of naked siTNF-α did not result in this suppression, the results indicate that combination with a carrier is important for the nose-to-brain delivery of siRNA and that our micelles act as carriers that promote nose-to-brain siRNA delivery.

**Figure 5:**
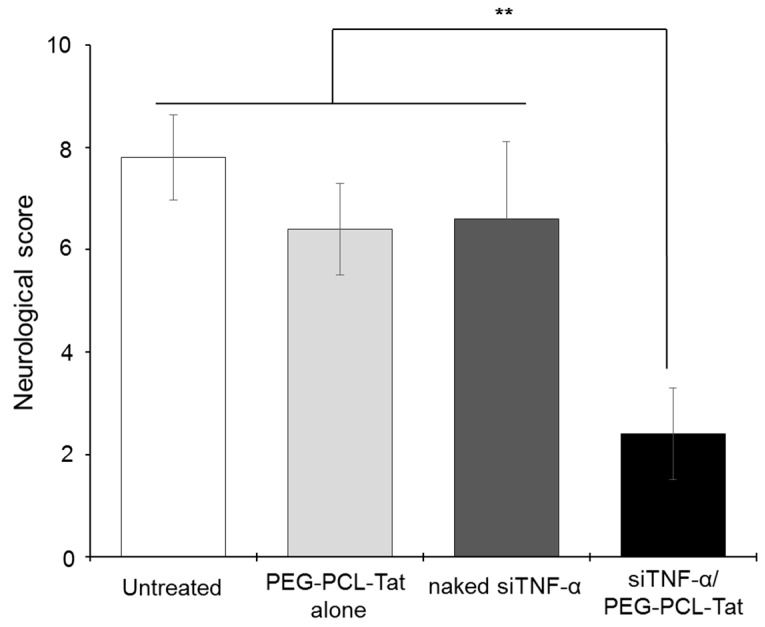
The neurological score of MCAO rats treated with intranasal administration of siTNF-α/PEG-PCL-Tat complex. The neurological score was evaluated based on spontaneous activity, drifting during displacement, parachute reflex, and resistance to right forepaw stretching. Each bar represents the mean ± S.E. (*n* = 5). ** *p* < 0.01.

Figure 5 shows the neurology score after 22 h of reperfusion in t-MCAO rats. The graph shows that the untreated group, as well as the PEG-PCL-Tat-only group and siRNA-only group, had a high neurology score, whereas the siTNF-α/PEG-PCL-Tat complex group showed a significant improvement in neurology score compared to the other groups.

The results above indicate that nasal administration of siTNF-α with PEG-PCL-Tat effectively suppresses the production of TNF-α in damaged brain tissue and significantly improves the symptoms of cerebral ischemic reperfusion injury such as the expansion of edema and infarcted area and the further worsening of nervous impairment.

## 4. Discussion

In this study, we focused on nose-to-brain delivery, which facilitates the transport of pharmaceuticals to the brain directly across the nasal mucosa, without going through the BBB. We selected siTNF-α, which has an anti-inflammatory effect, as a therapeutic, and we used PEG-PCL as a drug delivery carrier system along with the HIV-derived Tat peptide, which facilitates improved cell membrane permeability and bound them together to for PEG-PCL-Tat to be used in our research. To evaluate the effectiveness of the PEG-PCL-Tat complex on the nasal administration of siRNA to treat cerebral ischemic reperfusion injury in t-MCAO model rats, we loaded siTNF-α into the complex to create a siTNF-α/PEG-PCL-Tat complex. We administered this complex as well as only siTNF-α and only the PEG-PCL-Tat complex intranasally to t-MCAO model rats and conducted a comparative investigation on the treatment efficacy of siTNF-α/PEG-PCL-Tat complex administration against ischemic reperfusion injury.

The siTNF-α/PEG-PCL-Tat complex was formed by dripping a siTNF-α solution into a PEG-PCL-Tat solution and then letting it stand for 30 min. The complex formation rate was evaluated using a SYBR Green assay, which showed that the fluorescence intensity decreased as the N/P ratio increased, confirming that the complex formation rate was increased (Figure 1). In addition, the physical properties of MPEG–PCL-Tat/siRNA at various N/P ratios were evaluated (Table 1). The particle size of MPEG-PCL-Tat/siTNF-α complexes showed 50 to 100 nm, and the particle size decreased with an increase in the N/P ratio. This is because that MPEG–PCL-Tat can condense siRNA by electrostatic interaction with increase of the number of basic amino acids (arginine and lysine) in MPEG-PCL-Tat. This result is due to the fact that arginine has a strong condensing ability with siRNA due to its guanidinium group; therefore, MPEG–PCL-Tat at higher N/P ratio can condense them by increase of arginine. On the other hand, the zeta potential of these complexes increased with an increase in the N/P ratio. As shown in Table 1, at an N/P ratio of 5, the surface charge of the complex was decreased rather than MPEG-PCL-Tat alone, indicating that the basic amino acids of MPEG–PCL-Tat may be nearly neutralized by the interaction with negative charges in the siRNA. In addition, the zeta-potential of these complexes increased depending on the N/P ratio, and the surface charges of complexes with N/P ratios of 20 and 30 was over 15, indicating that arginine and lysine in MPEG–PCL-Tat appeared on the surface of the complexes at these N/P ratios. At N/P ratios up to 20, siRNA condensation occurred.

Subsequently, the cellular uptake ability of siRNA by MPEG-PCL-Tat were determined (Figure 2). the intracellular siRNA uptake of siRNA/MPEG-PCL-Tat complexes increased with an increase in the N/P ratio, and the complexes at an N/P ratio of 30 were seen to have the greatest intracellular uptake efficacy. We hypothesized that the number of free Tat peptides on the surface of MPEG–PCL-Tat complexes increased with the increase in the N/P ratio; therefore, the complexes with N/P ratios greater than 20 exhibited highly efficient cellular uptake because of the positive charge based on the arginine residues. In general, the internalization mechanism of Tat peptide was micropinocytosis [32]. We previously determined the cellular uptake pathway of pDNA by MPEG-PCL-Tat in detail using 4 °C transfection for endocytosis inhibition, a macropinocytosis inhibitor, a caveola-mediated pathway inhibitor, and a clathrin-mediated pathway inhibitor, and then, the cellular uptake activity markedly decreased in the presence of each inhibitor, particularly, macropinocytosis inhibitor and 4 °C significantly inhibited gene expression [30]. These suggest that basic amino acid tended to appear on the surface of the complex with the increase in the N/P ratio, and basic amino acid, especially the guanidine moiety, displayed on the surface of the complexes and enhanced the uptake by an endocytotic route, particularly macropinocytosis. 

Finally, in order to confirm the effectiveness of the PEG-PCL-Tat complex in the nasal administration of siRNA, t-MCAO model rats were prepared, and each treatment group was nasally administered the treatment for 30 min after ischemia. At 1.5 h after administration, blood was allowed to flow. At 22 h after reperfusion, the effect of the treatments against ischemic brain injury was evaluated via the area of infarcted regions, brain wet weight, TNF-α production, and neurology score.

Regarding the area of infarcted regions, each brain slice section had a widespread white-colored infarcted area in the untreated group (Figure 3). The siTNF-α-only group and PEG-PCL-Tat complex-only group also had the same result. In contrast, the siTNF-α/PEG-PCL-Tat complex group showed a reduced infarcted area in each specimen. A graph of the area of the infarcted region calculated using the image analysis program Image J confirmed that the siTNF-α/PEG-PCL-Tat complex group had a significantly reduced proportional area of the infarcted region compared to the other groups.

Based on the above results, it is thought that nasal administration of siTNF-α alone, which has therapeutic efficacy, leads to low absorption of the molecule in the nostrils and low transport to the brain, due to which it cannot demonstrate its therapeutic effect. We did not find any reduction in the infarcted area after administration of only the PEG-PCL-Tat complex, which suggests that the carrier itself does not have any effectiveness in reducing the infarcted area. However, we found that nasally administering the siRNA together with the high-molecular-weight cell-penetrating micelles reduced the infarcted area, suggesting that the PEG-PCL-Tat complex facilitated the efficient transport of siTNF-α to the brain, allowing the siRNA to demonstrate its anti-inflammatory effect.

Next, we measured the production of TNF-α. As shown in Figure 4, significantly suppressed production of TNF-α, a cytokine involved in inflammation in the infarcted areas, was observed in the siTNF-α/PEG-PCL-Tat complex group compared to the other groups. The neurology score, which expresses the degree of reperfusion injury, was high in the untreated group, siTNF-α-only group, and the PEG-PCL-Tat complex-only group but was comparatively low and significantly improved in the siTNF-α/PEG-PCL-Tat complex group. This shows that the reperfusion injury was clearly reduced.

Based on the above results, the siRNA-only group or the PEG-PCL-Tat complex-only group showed no improvements in the infarcted area proportion, TNF-α production, suppression of the worsening of brain weight, and neurology score. This is because siRNA-only is unable to penetrate the nasal mucosa and has poor in vivo stability, due to which it is broken down, and it is virtually impossible to transport it to its target location in the brain in time. As for the PEG-PCL-Tat alone group, the PEG-PCL-Tat itself is thought not to have any therapeutic efficacy. Whereas, the siTNF-α/PEG-PCL-Tat complexes markedly improvedthe infarcted area proportion, TNF-α production, suppression of brain weight worsening, and neurology score compared to the untreated group, PEG-PCL-Tat alone group, and naked siRNA group (Figure 5). Our previous study has already demonstrated that PEG-PCL-Tat could deliver the siRNA to the brain within 15 min (Olfactory bulb) to 60 min (whole brain) by intranasal administration through the olfactory nerve and trigeminal nerve pathway after across the nasal mucosal epithelial barrier [25,27], and further showed the therapeutic effects in rats with malignant glioma orthotropic implantation using anti-Raf-1 siRNA [26]. In addition, the cellular uptake of siRNA was markedly improved by using MPEG-PCL-Tat but never improved by siRNA alone (Figure 2), suggesting that the siTNF-α complexed with MPEG-PCL-Tat would suppress TNF-α in target cells in brain in vivo experiment using t-MCAO rats (Figure 4). Therefore, in this study, MEG-PCL-Tat would deliver the siTNF-α to the brain and target cells and lead to demonstrate the marked therapeutic effect for the ischemia-reperfusion injuries. This suggests that the PEG-PCL-Tat micelle is useful as a carrier for the nose-to-brain siRNA therapy for ischemia-reperfusion injuries.

## 5. Conclusions

In this study, we showed that it is possible to avoid the BBB and achieve efficient delivery of therapeutics to the brain by loading them onto a PEG-PCL-Tat complex and administering it nasally, which has the merits of being non-invasive and self-administrable with frequent doses. This suggests that this treatment method may facilitate drug delivery in patients with intractable brain diseases such as cerebral ischemic reperfusion injury and holds promise as an effective future treatment method for cerebrovascular disease. However, as the PEG-PCL-Tat does not have any selectivity to the ischemic tissue, the surface modification of the targeting moiety that increases the selectivity of PEG-PCL-Tat to ischemic environment area should be required for clinical use.

## Figures and Tables

**Table 1:** Particle size and zeta-potential of MPEG-PCL-Tat/siTNF-α complexes.

N/P Ratio	Particle Size (nm)	Zeta-Potential (mV)
0	32.1	7.26
5	105	5.35
20	60.6	16.56
30	62.4	19.42
