# Correction: Therapeutic Effects in a Transient Middle Cerebral Artery Occlusion Rat Model by Nose-To-Brain Delivery of Anti-TNF-Alpha siRNA with Cell-Penetrating Peptide-Modified Polymer Micelles. *Pharmaceutics*, 2019, *11(9)*, 478

**DOI:** 10.3390/pharmaceutics11120689

**Published:** 2019-12-17

**Authors:** Takanori Kanazawa, Takumi Kurano, Hisako Ibaraki, Yuuki Takashima, Toyofumi Suzuki, Yasuo Seta

**Affiliations:** 1 School of Pharmacy, Tokyo University of Pharmacy and Life Sciences, 1432-1 Horinouchi, Hachioji, Tokyo 192-0392, Japan; phta19001@g.nihon-u.ac.jp (T.K.); ibaraki@toyaku.ac.jp (H.I.); takasima@toyaku.ac.jp (Y.T.); setayas@toyaku.ac.jp (Y.S.); 2 School of Pharmacy, Nihon University, 7-7-1 Narashinodai, Funabashi, Chiba 274-8555, Japan

The appropriate information for the reprint used with permission from [2] is missing in our paper (Figure 2B) [1]. Therefore, the appropriate credit is inserted in the caption of Figure 2B as below. This insertion does not affect the scientific results. The manuscript will be updated, and the original article will remain online webpage (https://www.mdpi.com/1999-4923/11/9/478) with a reference to this correction.

## Figures and Tables

**Figure 2:**
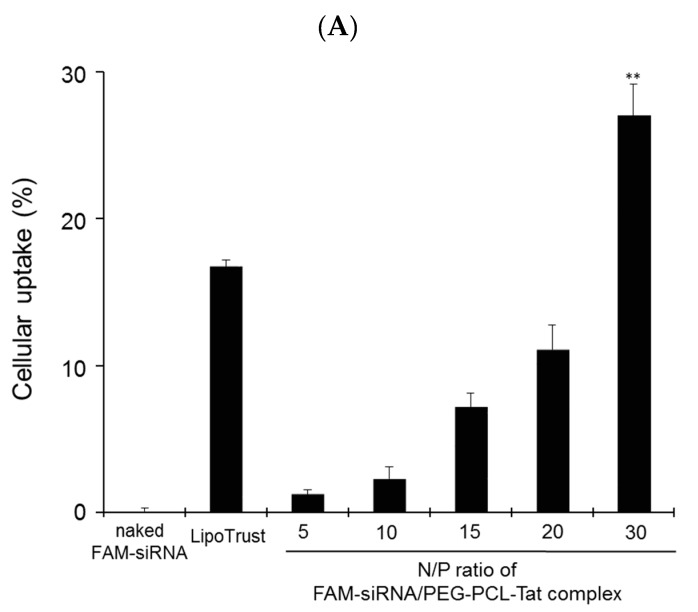

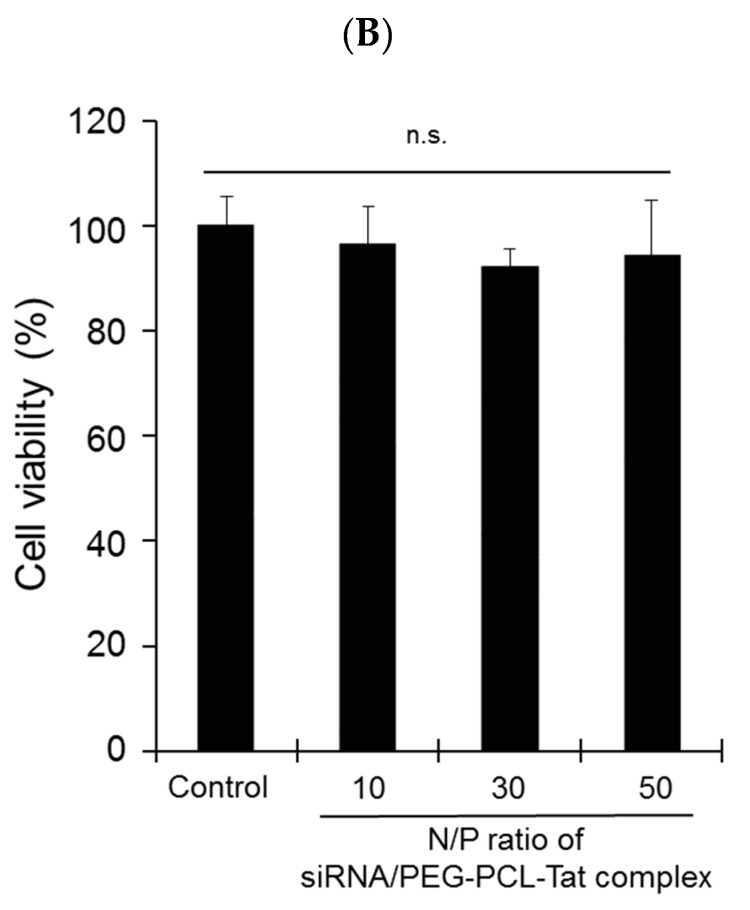
Cellular uptake of siRNA and cytotoxicity by PEG-PCL-Tat micelles in rat RN33B neuronal cells. RN33B cells were transfected with naked FAM-siRNA (1 μg), FAM-siRNA (1 μg) complexed with PEG-PCL-Tat (N/P ratio: 5–30), or Lipotrust as positive control. (**A**) After incubation for 4 h, the cellular uptake (%) of FAM-siRNA into RN33B cells was determined by flow cytometry. (**B**) Adapted with permission from [2]. Copyright 2019 American Chemical Society. After incubation for 3 h, in vitro cytotoxicity by PEG-PCL-Tat was determined by WST-8 assay. Each bar represents the mean ± S.D. (*n* = 4). **p < 0.01 vs. other groups, ^n.s.^*p* > 0.05.
